# The Strategy to Prevent and Regress the Vascular Calcification in Dialysis Patients

**DOI:** 10.1155/2017/9035193

**Published:** 2017-02-14

**Authors:** Nai-Ching Chen, Chih-Yang Hsu, Chien-Liang Chen

**Affiliations:** ^1^Department of Neurology, Kaohsiung Chang Gung Memorial Hospital, Chang Gung University College of Medicine, Kaohsiung, Taiwan; ^2^School of Medicine, Chung Shan Medical University, Taichung City, Taiwan; ^3^Division of Nephrology, Kaohsiung Veterans General Hospital, Kaohsiung, Taiwan; ^4^Department of Medicine, National Yang-Ming University School of Medicine, Taipei, Taiwan

## Abstract

The high prevalence of arterial calcification in end-stage renal disease (ESRD) is far beyond the explanation by common cardiovascular risk factors such as aging, diabetes, hypertension, and dyslipidemia. The finding relies on the fact that vascular and valvular calcifications are predictors of cardiovascular diseases and mortality in persons with chronic renal failure. In addition to traditional cardiovascular risk factors such as diabetes mellitus and blood pressure control, other ESRD-related risks such as phosphate retention, excess calcium, and prolonged dialysis time also contribute to the development of vascular calcification. The strategies are to reverse “calcium paradox” and lower vascular calcification by decreasing procalcific factors including minimization of inflammation (through adequate dialysis and by avoiding malnutrition, intravenous labile iron, and positive calcium and phosphate balance), correction of high and low bone turnover, and restoration of anticalcification factor balance such as correction of vitamin D and K deficiency; parathyroid intervention is reserved for severe hyperparathyroidism. The role of bone antiresorption therapy such as bisphosphonates and denosumab in vascular calcification in high-bone-turnover disease remains unclear. The limited data on sodium thiosulfate are promising. However, if calcification is to be targeted, ensure that bone health is not compromised by the treatments.

## 1. Introduction

Cardiovascular disease (CVD) is the leading cause of mortality and morbidity among patients with end-stage renal disease (ESRD) who are on chronic dialysis [1]. According to the US Renal Data System, CVD accounts for approximately 40% of mortality among patients on dialysis and is the main cause of hospitalization [2, 3]. Both traditional and nontraditional risk factors have been implicated in the development of CVD in chronic dialysis patients. Traditional risk factors are those used to predict coronary heart disease outcomes in the general population and include hypertension, smoking, hyperlipidemia, hyperglycemia, and obesity. Nontraditional risk factors (i.e., anemia, abnormal calcium/phosphorus metabolism, hyperhomocysteinemia, and malnutrition) are uremia-related factors that increase in prevalence as kidney function declines and contribute to the excess risk of CVD observed in patients with chronic kidney disease (CKD) [4].

Coronary artery calcification is much more prevalent in ESRD patients than in those without kidney diseases and contributes to extremely high morbidity and mortality. Recent evidence suggests that the interaction of traditional (i.e., age, smoking, diabetes mellitus [DM], hypertension, and dyslipidemia) and uremia-related so-called cardiovascular risk factors (e.g., hyperphosphatemia, high calcium × phosphorus product, oxidative stress, systemic inflammation, protein energy wasting, P-cresol, fetuin A, the osteoprotegerin (OPG)/receptor activator of NF-*κ*B (RANK)/RANK ligand system, and osteopontin) contributes to excessive and accelerated vascular calcification in CKD patients [5]. Intervention for these risk factors may delay the progression of vascular calcification. Cinacalcet with low-dose active vitamin D attenuated the progression of vascular and aortic valve calcification in 360 hemodialysis patients [6]. Kidney transplantation offers a means to restore kidney function and mineral metabolism at the same time. Removal of CKD-related risk factors through kidney transplantation may attenuate the rate of progression compared to those who remained on dialysis. Observation of kidney transplant recipients for 2.5–4.0 years revealed a progression of coronary artery calcification at a rate of 11% per year [7, 8].

These data suggested that vascular calcification, once it occurs, is unlikely to be reversed. Thus, therapeutic interventions that stop and reverse calcification may be of great value to patients with ESRD with vascular disease. Currently, no definite therapy has emerged. Hence, we reviewed the current strategy and treatments for vascular calcification in CKD patients.

## 2. Could the Extraosseous Calcification Be Reversed?

Can it reverse calcification? The answer is yes. Uremia-related vascular calcifications contain poor crystalline insoluble whitlockite and soluble amorphous calcium phosphate [9]. Although there was a portion of soluble amorphous calcium-phosphate in uremic related vascular calcification, the majority of the calcification is extremely insoluble whitlockite under physiological conditions that is difficult to mobilize the calcium deposits. However, vascular and soft tissue calcifications induced by calcitriol administration to rats partially revert after withdrawal of calcitriol treatment [10]. An active cellular process seems to be involved in regression of vascular calcification. High intake of vitamin K1 supplementation can result in regression of vascular calcification and restore the artery elasticity by virtue of its ability to activate matrix Gla protein, a local intravascular calcification inhibitor [11]. Furthermore, in clinical practice, extraosseous calcification such as tumor calcinosis and uremia-related vascular calcification such as calciphylaxis were considered controllable, at least partially, by parathyroidectomy and multiple intervention treatments including negative calcium and phosphate balance and avoiding calcification inducers and restoration of anticalcification factor balance [12–16].

## 3. Clinical Presentation of Calcification in CKD Patients

**Figure 1:**
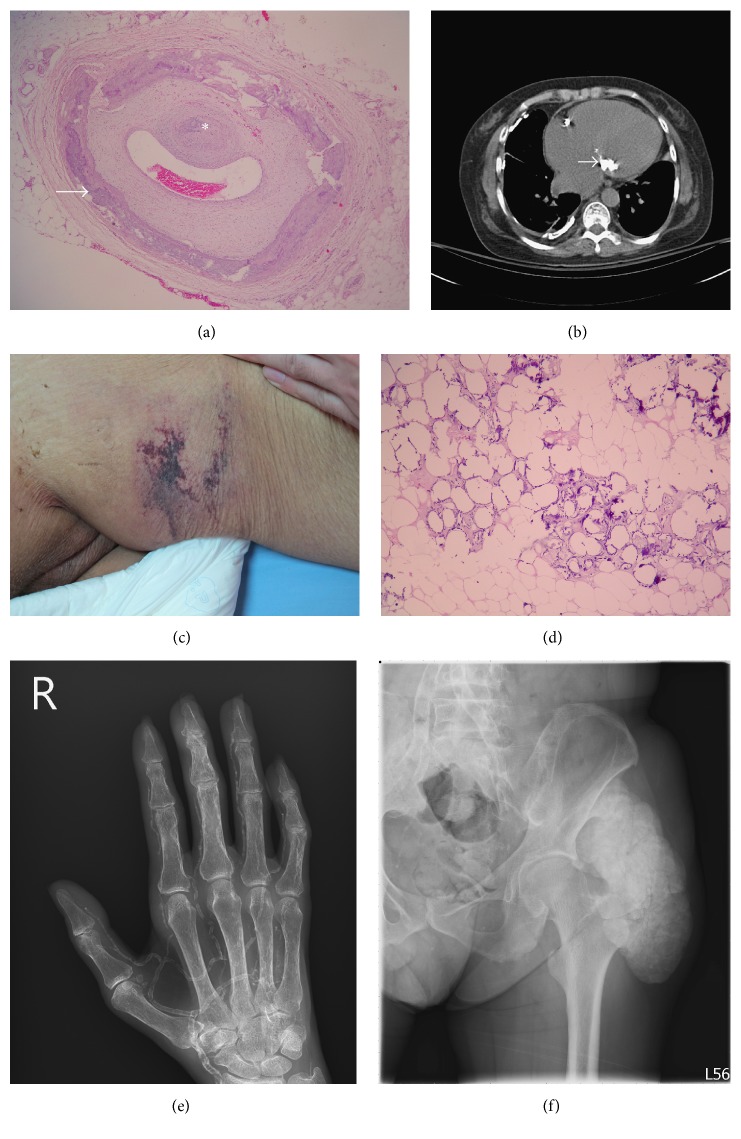
(a) Radial artery of a patient with end-stage renal disease showing intimal (*∗*) as well as medial calcification (arrow) under hematoxylin and eosin stain. (b) Calcifications can be seen on the mitral valve (arrow) in computed tomography studies. (c) Macroscopic evidence of calciphylaxis, (d) skin biopsy showing fat necrosis, composed of necrotic adipocytes, minimal inflammatory cell infiltration, and extensive calcification under hematoxylin and eosin stain, and (e) right hand radiographic evidence of severe heavy medial calcification of the radial arteries and their branches as shown by the so-called tram track phenomenon were found in a hemodialysis patient with calciphylaxis. (f) Left hip radiographs show a large radiopaque lesion on the soft tissue around the hip joint comprising multiple round calcified masses.

There are four different types of vascular calcification (Table 1) [17] and one type of soft tissue calcification: intimal artery calcification or medial artery calcification (Figure 1(a)), cardiac valve calcification (Figure 1(b)), calciphylaxis (Figures 1(c)–1(e)), and tumor calcinosis (Figure 1(f)). These four types are the consequence of distinct yet overlapping pathological mechanisms, and they are by no means mutually exclusive. The reliability of distinguishing medial from intimal calcification, in theory, is easy, based on light microscopic examination. On the radiography and ultrasonography images, intimal calcification is disclosed as irregular, discrete, plague-like calcification and medial calcification reveals tram-tract, nonstenotic diffuse calcified wall thickness. However, distinction has proved to be more difficult in clinical practice [18]. The contribution of intimal atherosclerotic calcification to plaque rupture complicated with obstruction is undefined. Medial artery calcification contributes to vascular stiffness, which increases pulse-wave velocity to decrease diastolic blood pressure and increase systolic blood pressure. Patients undergoing chronic hemodialysis who had intimal calcification had a higher relative risk of mortality than patients with medial calcification based on both X-ray examinations of the abdominal aorta and thigh arteries and ultrasonography of the common carotid arteries [19]. Cardiac valve calcification is significantly associated with calcification in the vascular bed, even after adjusting for traditional cardiovascular risk factors, possibly reflecting a predilection for ectopic calcification in certain subpopulations [20, 21]. Previous studies demonstrated significant correlation between valvular calcification and the coronary artery calcification score as detected by computed tomography in both ESRD and non-ESRD patients [22, 23]. The calcified regions of the cardiac valves share common features with arterial atherosclerotic plaques, with infiltration of inflammatory cells, calcium deposits, and bone matrix proteins, suggesting that valvular and vascular calcifications are likely associated syndromes [24–26]. Medial calcification develops concurrently with valve calcification and classical atherosclerosis, which is itself accelerated in a CKD-related inflammation state [27]. The synergistic effect could increase cardiovascular mortality.

## 4. How to Measure and Monitor Vascular Calcification

The evaluation of vascular calcification therapies is problematic because of lack of good methods to quantify it. Computed tomography of the aorta or coronary arteries is commonly used and is the only modality that can yield truly quantitative results. However, the cost is high and the process is not easy because the patients need to control their heart rate in order to evaluate coronary artery calcification. Clinical practice needs a simple technique to provide useful information. Radiography of the lateral abdomen (abdomen aorta) [28] or chest (aortic arch) [29] and the hand [30] can be used to detect the presence or absence of vascular calcification, and an echocardiogram can be used as a reasonable alternative to computed tomography-based imaging to detect the presence or absence of valvular calcification.

## 5. Strategy and Treatments for Vascular Calcification

Therapeutic interventions that stabilize or potentially reverse calcification may be of great value to patients with ESRD. Bone tissue has been detected in areas of vascular calcification, including osteoblast- and osteoclast-like cells, and in various bone-related extracellular matrix proteins, suggesting that the mechanisms of formation of mineralized vessels and bone are similar [26, 31]. However, if calcification is to be targeted systemically, caution must be exercised to ensure that bone and teeth health are not compromised. Growing evidence linking bone with different functional and structural characteristics of the arterial tree has contributed to the development of the concept of bone-vascular axis. Chemical mediators of bone metabolism such as matrix Gla protein, osteocalcin, bone morphogenetic protein, osteopontin, osteonectin, osteoprotegerin, receptor activator of nuclear factor kappa B ligand (RANKL), fetuin A, and inflammatory cytokines are also involved [32].

Although there was disappearance of a portion of uremic vascular calcification, the majority of vascular calcification that is composed of highly insoluble apatite is difficult to mobilize the calcium deposits in a short-time period. The strategies should combine the preventive and treatment approaches. The strategy goal is to reverse or stop the “calcium paradoxl;” that is, the lack of mineral in the bones makes them weak and excessive amount of calcium in blood vessels makes them more rigid [33]. Specific interventions in CKD patients without or with vascular calcification are aimed at restoring a new balance between pro- and anticalcification factors, at the same time considering the bone-vascular axis. In addition to traditional cardiovascular risk factors such as diabetes mellitus and blood pressure control, the strategies are as follows.

### 5.1. Minimize Inflammation

Recent evidence demonstrates that chronic inflammation, a nontraditional risk factor, is also commonly observed in ESRD patients. The causes of inflammation in ESRD are multifactorial, and while it may reflect underlying CVD, an acute-phase reaction may also be a direct cause of vascular injury by several pathogenetic mechanisms [34]. Many of the inflammatory markers and mediators such as interleukin 1 (IL-1), IL-6, C-reactive protein, and tumor necrosis factor alpha (TNF*α*) are found to promote vascular calcification in CKD patients [32, 34, 35]. The switch from cuprophane dialyzers to more biocompatible materials and ultrapure dialysate has made tremendous contributions to the lowering of inflammation in dialysis patients [36, 37]. A new dialysis membrane called the high cut-off dialyzer allows the elimination of molecules with a size of up to 45 kDa in chronic dialysis patients, which could reduce the procalcific effects of serum on vascular smooth muscle cell in vitro [38]. It was demonstrated that dietary phosphorus increased serum TNF*α* and malnutrition accelerated the progression of vascular calcification in uremic rats [39]. Furthermore, the introduction of high-dose iron preparations raises the future specter of inadvertent iatrogenic labile iron to accelerate early atherogenesis by increasing superoxide production and upregulating adhesion molecules [40, 41]. Thus, it could be speculated that adequate dialysis, appropriate dialyzer, use of ultrapure dialysate, avoiding malnutrition, and avoiding labile iron could improve inflammation in dialysis patients.

### 5.2. Maintain Appropriate Bone Turnover: Avoid Low and High Bone Turnover

CVD association with low- and high-bone-turnover disease is a biphasic relationship [42]. Under a high-bone-turnover status, the activation of osteoclast which stimulates bone resorption exceeds the bone formation by osteoblast activity through bone remodeling to release excessive calcium and phosphate from the bone into the extracellular fluid. Under a low-bone-turnover status, defective bone mineralization releases excessive calcium and phosphate into the extracellular fluid and causes vascular calcification [43]. Hence, how to keep appropriate bone turnover is very important. As mentioned earlier, bone biopsy is the gold standard for the diagnosis of bone turnover, but it is an invasive method and cannot be routinely performed. Radiographs and bone densitometry are not helpful for the diagnosis of adynamic bone disease. Low bone turnover may be suspected based on the results of biochemical parameters such as low parathyroid hormone (PTH) levels, for example, PTH levels less than twice the upper normal limit of a particular PTH assay or classic PTH levels of 150 pg/mL according to the previous Kidney Disease Outcomes Quality Initiative (KDOQI) American guidelines, a range that has a reasonably good predictive value [44]. The predictive value may be increased by adding low bone alkaline phosphatase (bAP) levels. The best cut-off for bAP to discriminate low from nonlow bone formation rate was 33.1 U/L and to discriminate high from nonhigh bone formation rate was 42.1 U/L [45]. In fact, many studies have found the bone formation rate to be better correlated with plasma bAP levels than with either plasma total alkaline phosphatase or PTH concentrations [46–49]. Despite its weaknesses as a biomarker, PTH represents perhaps the best current option for noninvasive assessment of bone turnover. Medical or surgical treatments to control hyperparathyroidism should be emphasized, and low-bone-turnover diseases should be avoided to maintain the intermediate PTH levels (i.e., 2–9 times the upper limit of normal for a particular PTH assay) of the patients, which is currently considered a desirable range according to the Kidney Disease Improving Global Outcomes (KDIGO) guidelines [46, 50].

#### 5.2.1. Low-Bone-Turnover Disease Treatment

Aluminum-based phosphate binders have continued to be used not only in Australia but elsewhere in the world, albeit less commonly in Europe and very little in North America [51]. Thus, as a first step, deferoxamine is considered important in the treatment of aluminum-induced forms of low-bone-turnover disease because it is easy to reverse with deferoxamine (5 mg/kg/week), which facilitates removal by dialysis. The vascular calcification of adynamic bone disease could be attributed to the low capacity of bones to accommodate a phosphate or calcium load in low-bone-turnover disease [43]. Vitamin D receptor activators restored osteoblast activity, increased the osteoid volume, and reduced intravascular calcium accumulation in adynamic animal models [52]. In clinical practice, the use of low calcium dialysate [53] and native vitamin D supplementation to achieve calcidiol levels of 20–30 ng/mL should be considered [54, 55]. Furthermore, antiresorption therapy, large doses of active form vitamin D, and high calcium dialysate should be avoided in these patients [55]. Recombinant PTH [56] and antisclerostin monoclonal antibodies as bone-stimulating agents [57] may be beneficial in these patients in the future. However, there are shortages of well-controlled clinical trials and limit to their use in the current period.

*Treatment of Low-Bone-Turnover Disease [44, 46, 55, 58]*

  Treatment of aluminum bone disease
  Avoid antiresorptive agents such as bisphosphonates and denosumab
  Avoid PTH oversuppression due to calcimimetics or excessive use of the active form of vitamin D
  Avoid high calcium dialysate
  Avoid excessive calcium-based phosphate binder
  Consider using the following: noncalcium, non-aluminum-based phosphate binders; native vitamin D to achieve calcidiol levels of 20–30 ng/mL; low calcium dialysate (1.25 mmol/L); recombinant PTH and antisclerostin monoclonal antibodies

#### 5.2.2. High Turnover Bone Disease Treatment

Patients with more than 9 times the upper limit of normal for a particular PTH assay are considered to have high-bone-turnover disease, according to the KDIGO guidelines [46]. Bone biopsy should always be performed before hyperparathyroidism treatment if there is a marked difference between the bone metabolism marker values and the serum P, Ca, and iPTH levels that could be estimated from it or if osteomalacia is suspected because of a history of heavy exposure to aluminum and bone pain or fracture of unknown cause [44, 46, 58]. The addition of an active vitamin D analogue is appropriate for uncontrolled PTH. The selection of the best vitamin D analogue remains the subject of controversy owing to their selective ability to result in less positive calcium and phosphate balance, which are key mediators of vascular calcification [59]. Further basic and clinical investigations are needed to determine the optimal doses and type of active D analogue or dual therapies with vitamin D therapy (nutritional vitamin D plus active form or vitamin D analogue). International guidelines, including the KDOQI American commentary and the 2009 KDIGO guideline, suggest that the primary choice of a hyperparathyroidism treatment may be based on serum calcium and phosphate. Cinacalcet could markedly improve the achievement of target levels in patients with secondary hyperparathyroidism [60]. Furthermore, the efficacy of cinacalcet in preventing the progression of CV calcifications (ADVANCE) trial suggested that cinacalcet plus low doses of vitamin D may attenuate the progression of vascular calcification [6]. Parathyroid treatment including percutaneous ethanol parathyroid injection and parathyroidectomy, which is reserved for severe secondary hyperparathyroidism, could increase serum osteoprotegerin and fetuin A levels to restore anticalcification factor balance and negative extraosseous calcium and phosphate balance in order to reduce or stabilize vascular calcification [61, 62].

*Treatment of High-Bone-Turnover Disease [44, 46, 58]*

  Always consider bone biopsy before hyperparathyroidism treatment if there is a marked difference between the bone metabolism marker values and the serum P, Ca, and iPTH levels that can be estimated from it or if osteomalacia is suspected because of a history of heavy exposure to aluminum or bone pain or fracture of unknown cause
  Always consider phosphate/calcium control more than parathyroid gland control
  Use native vitamin D to achieve calcidiol levels above 30 ng/mL
  Use activated vitamin D/vitamin D analogue alone or with cinacalcet
  Parathyroid intervention (parathyroidectomy or percutaneous ethanol injection therapy) should be considered if a high blood PTH level (>800 pg/mL) remains resistant to medical treatment and hypercalcemia or hyperphosphatemia is also noted.

### 5.3. Avoid Calcium Positive Balance

The related concept that Ca concentrations do not reflect balance should always be kept in mind during clinical practice [42, 63, 64]. Ca balance can be negative, neutral, or positive, depending on treatment with calcium salts, vitamin D, and Ca levels in the dialysate. In dialysis patients with low bone turnover whose plasma PTH levels are <150 pg/mL (16.5 pmol/L) and with preexisting severe vascular and/or other soft tissue calcifications, negative calcium balance was considered. Non-calcium-based phosphate binders with 1.25 mmol/L calcium dialysate are suggested. In dialysis patients without cardiovascular complication, neutral calcium balance was considered, and the limit Ca-based binder is set at 1.5 g elemental Ca per day [44, 46]. In addition, dialysate calcium concentration should be viewed as part of the source of positive calcium balance, as an inlet dialysate Ca concentration of 1.75 mmol/L leads to a positive Ca positive balance and possibly a negative calcium balance when dealing with a Ca dialysate of 1.25 mmol/L [65, 66]. Mild and asymptomatic hypocalcemia (e.g., in the context of calcimimetic treatment and parathyroidectomy) can be tolerated in order to avoid inappropriate calcium loading in adults [67, 68]. The increased acceptable idea is that the vascular calcification of chronic kidney disease patients should influence the choice of the negative calcium balance by non-calcium phosphate binder and 2.5 mEq/L dialysate calcium concentration as more than biochemical serum calcium levels.

### 5.4. Avoid Phosphate Positive Balance

The total body content of P results from (1) dietary intake, (2) gastrointestinal absorption, and (3) kidney and stool excretion. When renal function deteriorates, positive phosphate balance is inevitable. Based on epidemiological data linking hyperphosphatemia to reduced survival, the KDOQI guidelines suggested that P levels should be maintained between 3.5 and 5.5 mg/dL in dialysis patients [44]. However, KDIGO guidelines have a new suggestion to maintain P levels toward the normal range in ESRD [46]. Patients whose plasma phosphate rapidly refills from intracellular stores as dialysis removes it may respond well to increasing dialysis time with standard thrice-weekly dialysis or changing to nocturnal dialysis, either daily or thrice weekly [69]. Altering dialysis membranes and blood and dialysate flow rates have very limited effects, although hemodiafiltration appears to have a modest benefit in removing phosphate by convection [69–72]. Intestinal phosphate absorption can also be reduced by limiting or discontinuing activated vitamin D therapy. Activated vitamin D analogues, particularly paricalcitol, appear to be less phosphatemic. Cinacalcet improves the attainment of KDOQI bone metabolism serum phosphate levels in dialysis patients with various stages of secondary hyperparathyroidism [73]. The use of certain high-protein low-phosphate foods such as whey protein and egg whites can also help control phosphate levels without the threat to nutritional status presented by restriction of the usual protein-linked sources of dietary phosphate [74, 75]. Phosphate is usually present in food such as beverages and compounds that contain inorganic phosphorus, which is frequently used in the food industry to extend shelf life, enhance flavor, and improve the color of food products [72–74]. The other positive vascular phosphate balance concerns including hyperparathyroidism leading to phosphate efflux from bone [73]. In addition to adequate dialysis clearance and phosphate diet control, pharmacological phosphate binder treatments should be considered. Calcium-free-based phosphate binder could decrease mortality. Sevelamer has pleiotropic effects on lipid profiles, fibroblast growth factor 23, inflammation, uremic toxins, oxidative stress, and fetuin A and improves endothelial dysfunction. Ferric citrate could provide another iron source and avoid intravenous iatrogenic labile iron, which accelerates early atherogenesis by vessel inflammation. Lanthanum carbonate has the most potent phosphate-binding effects in decreasing pill loading. Choosing the appropriate phosphate binder depends on the budget, pill load, beneficial additional pleiotropic effects, and side effects (Table 2) [40, 41, 76–80]. A flexible sequential approach to treatment may result in greater success to avoid phosphate positive balance.

### 5.5. Correction of Vitamin D and Vitamin K Deficiency

KDIGO recommends the correction of vitamin D insufficiency in CKD patients (i.e., 25(OH) D level, <20 ng/mL) [44]. There is some evidence that 25(OH) D supplementation can assist with the management of secondary hyperparathyroidism and possibly prevent vascular calcification [52, 81].

Vitamin K (K1 and K2) is involved in the production of bone and matrix amino acid *γ*-carboxyglutamic acid proteins, anticalcification, and bone-forming molecule. Low vitamin K concentrations increase the risks of bone fracture and vascular calcification. Experimental data suggest that vitamin K antagonist may decrease the activity of matrix-g-carboxyglutamic acid protein, a strong inhibitor of soft tissue calcification [82]. The use of vitamin K antagonists is suggested in patients with tissue calcification [83, 84]. A subgroup analysis of participants who were ≥85% adherent to a 500 *μ*g daily vitamin K1 treatment showed a lower CAC progression in the phylloquinone group than in the controls [85]. Vitamin K supplementation may be a simple means to prevent the progression of vascular calcification in hemodialysis patients, a population characterized by severe functional vitamin K deficiency [86]. It is hoped that the existing coronary artery calcification in dialysis patients in current trials be reduced.

### 5.6. Other Antiresorption Therapies

#### 5.6.1. Bisphosphonates and Denosumab

Bisphosphonates are synthetic analogues of inorganic pyrophosphate that have the ability to inhibit osteoclast-mediated resorption and inhibit calcium phosphate crystal deposition in the bone and vessels. In the search for a common mediator capable of influencing bone remodeling and vascular calcification, the OPG/RANK/RANK ligand system has received recent attention. Restoring a balanced RANKL-to-OPG by modulating OPG production may represent a therapeutic strategy to preventing bone loss and vascular calcification. Denosumab is considered as OPG mimicker. Bisphosphonates and denosumab could reduce vascular calcification in chronic kidney failure animal models and some case reports [87–90]. Antiresorption therapy could reduce bone turnover and should not be used in patients with adynamic bone diseases. Similar to bisphosphonates, denosumab should not be used in patients with adynamic bone disease [91, 92].

Bisphosphonates are cleared by the kidney and should be used with caution in patients with glomerular filtration rate (GFR) less than 30 mL/min. With lower GFR, there is increased drug accumulation in bone mineral and a theoretical risk of inducing adynamic bone disease. The decision to use a bisphosphonates in a CKD patient should be individualized per patient.

Denosumab, as bisphosphonates, inhibits osteoclast-mediated bone resorption, but because it is not cleared by the kidney, there is no concern of its accumulation in patients with CKD. However, several studies in CKD patients have shown that severe and life-threatening hypocalcemia can occur; thus, frequent monitoring of serum calcium is required [93–95]. In view of the potential role of the OPG-RANK-RANKL axis in the development of vascular calcification, whether this therapeutic compound can regress vascular calcifications is worth investigating. However, well-controlled trials are needed to confirm the benefit.

#### 5.6.2. Sodium Thiosulfate

Calciphylaxis involving arteriolar media calcification with ischemia, necrosis, and skin ulcerations could lead to 80% mortality [96]. Evidence suggests that disorders in the imbalance of the deficiencies of other calcification inhibitors are causally implicated in pathological calcification processes in the body [97]. Sodium thiosulfate, used as an antidote for cyanide poisoning, binds with vascular calcium salts to form a highly soluble calcium thiosulfate salt. The use of sodium thiosulfate in the treatment of calciphylaxis has been described primarily in case reports with partial-to-complete resolution of skin lesions. Sodium thiosulfate is generally well tolerated. Sodium thiosulfate is cleared by dialysis and, thus, doses need to be infused after each dialysis (25–50 g intravenously over 1 h). The most common adverse effects during sodium thiosulfate treatment for calciphylaxis were nausea, vomiting, and increased anion gap acidosis [98–101]. However, the current bicarbonate dialysate may resolve sodium thiosulfate-induced metabolic acidosis.

## 6. Conclusion

Although some experimental targets involved in calcium deposition are emerging, no intervention has been described to reliably reverse vascular calcification. Consistent implementation of a systematic multi-interventional treatment strategy is suggested, consisting of trigger-agent cessation (calcium-based phosphate binders, excessive activated vitamin D, and vitamin K antagonist) and supplemented by minimization of inflammation, control of bone turnover, and avoidance of positive calcium and phosphate balance. Correction of vitamin D and K deficiency or antiresorption therapy in high-bone-turnover disease and intravenous sodium thiosulfate to keep extraosseous calcium and phosphate negative balance as much as possible may alter the course of vascular calcification. Further studies are needed to confirm clinical effects.

## Figures and Tables

**Table 1:** Types of vascular calcification.

Type	Characteristics/risk factors	Complication
Atherosclerotic intimal calcification	Calcification of atherosclerotic plaques; eccentric lumen deformation by patchy calcification of the intima in the vicinity of lipid or cholesterol deposits as present in plaque calcification; patch or striped calcification on X-ray examination. Risk factors include hypercholesterolemia, metabolic syndrome, diabetes, and hypertension.	Ischemia/infarction
Arterial medial calcification	Calcification of the media in the absence of such lipid or cholesterol deposits, known as Mönckeberg-type atherosclerosis; tram-like or pipe calcification by X-ray examination. Risk factors include abnormal calcium-phosphate metabolism and inflammation.	Systolic hypertension, left ventricular hypertrophy
Heart valve calcification	Calcification of aortic valve or mitral valve leaflets as a consequence of abnormal calcium-phosphate metabolism, inflammation, and traditional cardiovascular risk factors such as hypercholesterolemia, metabolic syndrome, diabetes, and hypertension.	Heart failure
Calcific uremic arteriolopathy	Dermal arteriolar medial calcification and dermal fat necrosis, usually in the abdomen, thighs, breasts, and buttocks. X-ray examination of the extremities including the hands and feet reveals calcified artery in the absence of thrombosis. Risk factors include diabetes, obesity, vitamin K antagonist, and steroid.	Painful nodule and subcutaneous skin/fat necrosis wound

**Table 2:** How to choose phosphate binders depends on the budgets, pill loading, beneficial effects and side effects.

Phosphate binders	Relative coefficient	Pill burden	Beneficial effects (pleiotropic effects or others)	Side effect	Cost
Aluminum hydroxide	1.5	Low	No	Bone accumulation	Low
Calcium carbonate	1.0	High	No	Vascular and soft tissue calcification	Low
Calcium acetate	1.0	High	No	Vascular and soft tissue calcification	Low
Sevelamer carbonate/Sevelamer hydrochloride	0.75	High	Pleiotropic effects (lipid profiles, fibroblast growth factor 23, inflammation, uremic toxins, oxidative stress, fetuin A, and improvement of endothelial dysfunction)	Decrease absorption of vitamins A, D, E, and K; sevelamer hydrochloride (metabolic acidosis)	High
Lanthanum carbonate (Fosrenol® chewable tablet)	2.0	Low	No	Bone accumulation	High
Magnesium carbonate	1.7	High	No	Hypermagnesemia	Low
Fe-citrate (Nephoxil®)	1.14	High	Supply oral iron	Iron overload/diarrhea?	High
